# Critical review of OCT in clinical practice for the assessment of oral lesions

**DOI:** 10.3389/fonc.2025.1569197

**Published:** 2025-05-08

**Authors:** Fariba Esperouz, Domenico Ciavarella, Mauro Lorusso, Andrea Santarelli, Lorenzo Lo Muzio, Giuseppina Campisi, Lucio Lo Russo

**Affiliations:** ^1^ Department of Clinical and Experimental Medicine, University of Foggia, Foggia, Italy; ^2^ Department of Clinic Specialistic and Stomatological Sciences, Polytechnic University of Marche, Ancona, Italy; ^3^ Department of Biomedicine, Neurosciences and Advanced Diagnostics (Bi.N.D.), University of Palermo, Palermo, Italy

**Keywords:** OCT, OSCC, OPMD, limitation, AI

## Abstract

**Background:**

Optical Coherence Tomography (OCT) is an advanced imaging technique that is widely used in ophthalmology and is increasingly being applied in other fields of medicine. In oral oncology, OCT offers high-resolution, non-invasive (uses non-ionizing light), label-free, real-time imaging, providing detailed insights into tissue microanatomy and cellular structures, thus having the potential to improve early detection, monitoring and cost-effective screening of high-risk populations. However, significant challenges remain in applying OCT to OSCC and OPMDs, particularly in clinical practice.

**Methods:**

A comprehensive search of PUBMED, SCOPUS, and Web of Science databases was performed up to October 2024. Additional manual searches were conducted by screening article bibliographies. Inclusion criteria encompassed studies published in English involving human subjects and evaluating the role of OCT in OSCC and OPMD assessment, OCT utilization for margin resection, and artificial intelligence (AI)-assisted interpretation of OCT images. After removal of duplicates and screening of titles and abstracts, full-text analysis was conducted on eligible studies.

**Results:**

The technique has been investigated for its accuracy in identifying malignant changes in tissues before surgery and/or evaluating resection margins during surgery. Although early studies, primarily in animal models, have been extended to humans and have demonstrated the potential of OCT to accurately assess resection margins and identify precancerous lesions, significant limitations persist. The high cost of OCT equipment reduces its accessibility, availability and widespread use as a common investigation methodology in non-experimental settings. In addition, there is significant heterogeneity in the methodologies used to interpret OCT data, which is strictly operator dependent and may affect standardization and reproducibility of results. This is further complicated by the introduction and increased trend to adopt artificial intelligence (AI) algorithms in imaging evaluation. Machine learning and deep learning algorithms have shown superior diagnostic sensitivity and accuracy compared to clinician judgment. However, especially when used to assess resection margins, these algorithms may be significantly affected by sample extension and preparation, which remains a barrier to the routine clinical application of OCT systems.

**Conclusion:**

Addressing the advantages and challenges of this emerging technique may help focus future research on standardizing application protocols and enhancing AI-assisted analysis to improve diagnostic performance and facilitate clinical translation.

## 1 Introduction

Oral squamous cell carcinoma (OSCC) is a significant global health problem affecting approximately 390,000 people worldwide (1). It is the most common type of head and neck cancer and its incidence has been increasing recently, accounting for almost 90% of all oral cancer cases (2). Although the oral cavity is anatomically accessible, making mucosal assessment relatively straightforward, OSCC diagnosis is often delayed, leading to persistently high rates of prevalence, incidence and mortality (3). Some patients may develop Oral Potentially Malignant Diseases (OPMDs) prior to tumor onset. These conditions, which are associated with an increased risk of cancer, present with a range of clinical manifestations, including red, white or ulcerated areas (4), with either exophytic or endophytic growth patterns (5). Larger lesions may have heterogeneous patterns with deeper areas, which can be difficult to assess during initial biopsy procedures, potentially requiring multiple biopsies and causing patient discomfort.

Currently, histological examination of biopsy samples is used for assessment and surgery remains the primary treatment for most cases (6). To minimize the need for multiple surgeries, the integration of non-invasive preoperative techniques into clinical practice could improve lesion characterization and facilitate better decision making in biopsy site selection and initial curative surgery (7). Improved preoperative knowledge of the microscopic features of the lesion could help surgeons to refine surgical protocols, determining both the extent of surgery required and a more accurate understanding of histopathological details.

Imaging techniques such as computed tomography (CT) and magnetic resonance imaging (MRI) are used for this purpose. MRI, in particular, provides excellent soft tissue contrast without exposure to ionizing radiation. However, MRI has significant drawbacks, including long waiting times - typically 65 to 105 days (8) - and high costs, requiring specialized facilities. In addition, around 10% of patients cannot undergo MRI due to factors such as metal implants or claustrophobia (9). These limitations have restricted the widespread use of MRI in the preoperative evaluation of patients with suspected OSCC and OPMDs.

Widely recognized in ocular diagnostics, Optical Coherence Tomography (OCT) is increasingly applied in fields like dermatology, cardiology, odontology, gastroenterology, and oncology (10–12); Now, OCT’s use has been extended to the detection of early epithelial changes in head and neck, providing high-resolution images of tissue microanatomy and cellular structures of the mucous membranes (13, 14). This technique offers label-free, non-contact *in vivo* microscopy by utilizing non-ionizing visible light to analyze tissue optical properties (15–17) Employing a low-coherence broadband near-infrared light source, OCT achieves high spatial resolution (approximately 20 μm) and provides real-time imaging (18). The method is quick, repeatable, and well-tolerated by patients (19).overcoming the limitations associated with other imaging techniques such as computed tomography (CT) and magnetic resonance imaging (MRI). As a non-invasive, real-time imaging method, OCT provides valuable information suitable for assessing Oral squamous cell carcinoma (OSCC) and Oral potentially malignant diseases (OPMDs) and assists surgeons in evaluating resection margins (16).

Despite the numerous advantages mentioned above, a review of recent literature highlights several critical challenges associated with its clinical application. Therefore, the primary aim of this review is to highlight the challenges associated with the potential application of this technique in clinical practice.

## 2 Materials and method

A comprehensive search of PUBMED, SCOPUS, and Web of Science was performed up to October 2024, supplemented by manual searches of article bibliographies. Studies in English involving human subjects and evaluating the role of OCT in assessing OSCC and OPMD, the use of OCT in margin resection, and the application of AI for diagnosis using OCT were included.

## 3 Results

### 3.1 Study selection

**Figure 1:**
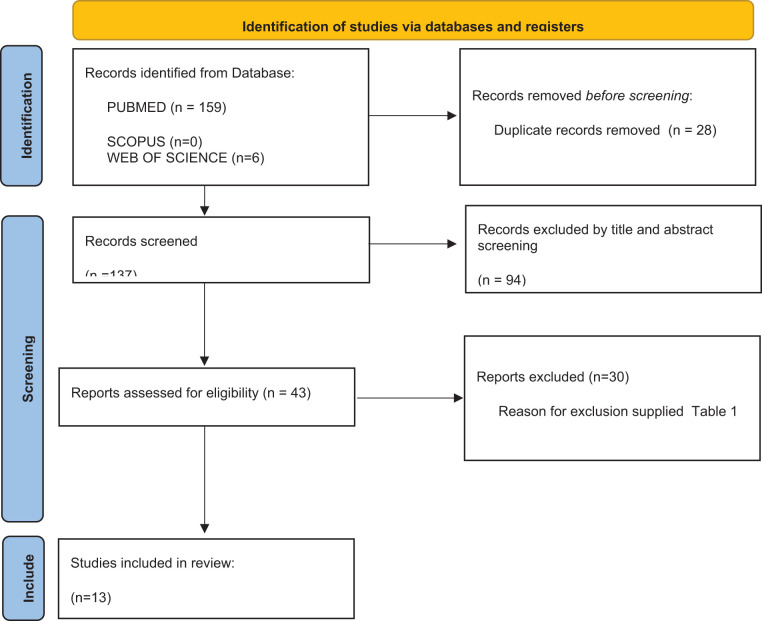
Flowchart of studies’ selection process.

The electronic search retrieved a total of 165 articles from the electronic database search up to October 2024. After the removal of duplicates (28), title and abstract analysis was performed on 137 articles, of which 94 were excluded after the screening. Full text analysis was performed on the remaining 43 articles, and 30 articles were further excluded. The final review includes 13 articles (12, 16, 17, 20–29) (**Figure 1**).

### 3.2 Summary of included studies

The eligible studies (**Table 1**) were conducted from 2008 to 2024, in various countries around the world, including: China (12, 27, 28), India (21–24), Italy (29), Malaysia (23), Taiwan (22–25), UK (16, 17) and USA (20–26).

**Table 1:** Elegibile studies.

ID	YEAR	COUNTRY	STUDY DESIGN	LESION TYPE	SAMPLE SIZE	SITE	STUDY DESIGN	STAGING	EDITION	NUMBER OF PARTICIPANTS	GENDER	MEAN AGE	MODEL
*Hamdoon*	2015	UK	Prospective study	OSCC	28	Ventro-lateral tongue (7), Floor of mouth (6), Retromolar trigone (4), Buccal mucosa (3), Lower lip (2), Hard palate (2), Upper lip (2), Soft palate 2.	Ex-vivo	T1 (20) T2 (8)	NR	28	F=9; M= 19	61	Michelson Diagnostics EX1301 OCT Microscope V1.0
*Heidari* (20)	2020	USA	Prospective study	OSCC	7	NR	*In vivo*	NR	NR	7	NR	NR	VCSEL
*James* (21)	2021	India	Prospective study	OPMD	347	Buccal mucosa, Labial mucosa	*In vivo*	NR	NR	232	NR	32	SD-OCT
*Jerjes* (17)	2019	UK	Prospective study	OSCC	60	Lateral tongue (15),Floor of the mouth (13), Ventral tongue (6),Lower Lip (5), Hard palate (5),Soft palate (4), Alveolar mucosa (4).	Ex-vivo	T1 (43) T2 (17)	NR	60	F=20; M=40	63	Michelson Diagnostics EX1301 OCT Microscope V1.0)
*Lee* (22)	2012	Taiwan	Observational	OSCC	137	NR	*In vivo*	NR	NR	54	NR	NR	(Santec, HSL-2100-ST)
*Obade* (23)	2021	Malaysia	Prospective study	OSCC	42	Tongue (16),Gingiva (9), Buccal mucosa (11), Lip (8).	Ex-vivo	NR	NR	44	NR	NR	Thorlabs OCS1300SS, USA
*Panzarella* (30)	2024	Italy	cross sectional	OSCC OPMD	21	Oral Leukoplakia (7), Oral Lichen Planus (7), OSCC (7)	*In vivo*	NR	NR	21	NR	NR	OCT SS-OCT VivoSight^®^, Michelson Diagnostics
*Sunny* (24)	2019	India	Prospective study	OSCC	125 (images)	Maxillary GBS (2),Buccal mucosa (6), Retro molar trigone (1), Maxillary alveolus (1),Mandibular alveolus (2), Tongue (2).	*In vivo*	T1 (1) T2 (5) T3 (1) T4a (2) T4b (5).	NR	14	F=4; M=10	55	SD-OCT
*Tsai* (25)	2008	Taiwan	Observational	OSCC	84	NR	*In vivo*	NR	NR	32	NR	30–77 years	SS-OCT
*Wilder- Smith* (26)	2009	USA	Prospective study	OSCC	50	NR	*In vivo*	NR	NR	50	NR	NR	NR
*Yang*	2021	China	Prospective study	OSCC (14); OPMD(5)	71	NR	Ex-vivo	NR	NR	19	F=9; M=10	61	Mach–Zehnder
*Yuan*	2021	China	Case-control	OSCC	100 resected patches as training set 29 resected patches are tested	Tongue (C:44; c:15) Gingiva (C:25;c:8) Cheek (C:21;c:2) Mouth floor (C:8;c:3)	*In vivo*	NR	NR	37	F=14; M=23	58.2	NR
*Zhou* (12)	2024	China	Observational	OSCC	18	Tongue	Ex-vivo	NR	NR	NR	NR	NR	SD-OCT

Ten studies evaluated OSCC lesions (12, 16, 17, 20, 22–26, 28), one evaluated OPMD (21), and two evaluated both (27, 29).

In five studies (12, 16, 17, 23, 27) OCT was used ex-vivo, in the remaining eight (20–22, 24, 26, 30–32), it was used *in-vivo*.

The OCT data were interpreted in two different ways, by the use of AI (20, 21, 24, 27, 32) or by clinicians (12, 16, 17, 22, 23, 26, 30, 31) (**Table 2**).

**Table 2:** Characteristic of OCT device.

ID	MODEL	FREQUENCIES (kHz)	WAVELENGTH (NM)	AXIAL RESOLUTION (µm)	LATERAL RESOLUTION (µm)	IMAGINE DEPHT (mm)
*Hamdoon*	Michelson Diagnostics EX1301 OCT Microscope V1.0	NR	1310 nm	<10µ m	<10µ m	1 mm
*Heidari*	VCSEL	200 kHz	1310 nm		NR	NR
*James*	SD-OCT	20kHz	930 nm	7 µm	15 µm	0.2–1 mm
*Jerjes*	Michelson Diagnostics EX1301 OCT Microscope V1.0)	>1 Hz	1310 nm	<10µm	<10µm	1 mm
*Lee*	Santec, HSL-2100-ST	NR	1310 nm	6 μm	NR	1 mm
*Obade*	Thorlabs OCS1300SS, USA	NR	1325 nm	NR	NR	NR
*Panzarella*	OCT SS-OCT VivoSight^®^, Michelson Diagnostics	NR	150 nm	<10µm	<7.5 µm	2 mm
*Sunny*	SD-OCT	1.2 kHz	930 nm	7.0 µm	15 µm	NR
*Tsai*	SS-OCT	20 kHz	1310 nm	8 µm	15 µm	1 mm
*Wilder- Smith*	NR	NR	NR	NR	NR	NR
*Yang*	Mach–Zehnder	100 kHz	87 nm	14 μm	17 μm	NR
*Yuan*	NR	NR	800 nm	1.5 μm	NR	NR
*Zhou*	SD-OCT	NR	840 nm	5 μm	15 μm	NR

## 4 Discussion

### 4.1 Limitations in oral clinical practice

The use of Optical Coherence Tomography (OCT) in oral pathology can be traced back to 2004, with studies conducted by Wilder-Smith, Chen, and Kim (10, 33–35); these early investigations focused exclusively on animal models.

Based on the analysis of the studies available in the literature, screened according to the following inclusion criteria: studies published in English; cohort, case-control, retrospective observational, or longitudinal study designs; studies investigating the role of OCT in the assessment of OSCC and OPMD; and studies conducted on human subjects (**Table 1**). The first human studies using OCT were not conducted until 2006, possibly due to the high cost of the technology. A commercial OCT system can cost between $40,000 and $150,000, making it generally available only in hospitals (36); It is therefore essential to investigate the development of low-cost OCT systems to enable the application of this technology in large-scale population screening. The availability of affordable, portable, and user-friendly OCT systems will be crucial to facilitating its widespread clinical adoption (36).

The *in vivo* study by Ridgway et al. (37) showed that while OCT can visualize the most superficial epithelial layers, its ability to image the basement membrane and subsurface structures is significantly limited. Moreover, image quality heavily depends on the operator’s technique (38),making histological examination necessary for a more complete analysis.

The analysis of oral mucosa images obtained by OCT allowed the distinction between normal and moderately dysplastic or mildly dysplastic mucosa was analyzed in the study conducted by Lee et al. (22), with a sensitivity of 82% and a specificity of 90%, allowing near real-time diagnosis of precancerous conditions by OCT imaging. The same level of accuracy (89.6%) was also found in the study *ex-vivo* by Obade (23), who noted that the ability of OCT to observe the integrity of the basement membrane is a key parameter in detecting OSCC and differentiating OSCC from oral dysplasia or benign conditions. Both Wilder-Smith (26) and Tsai (25) reported an accuracy exceeding 90% (93.10% and 100%, respectively) when comparing OCT data with histological findings, highlighting OCT is potential as a valuable tool for the early detection and diagnosis of oral lesions. Similar results were observed in a more recent study (12), which found that the mean grey value (MGV) of OSCC in OCT images was significantly higher than that of the surrounding healthy tissue. This suggests that MGV could be a useful parameter for distinguishing tumors from normal tissue.

Moreover, in the studies conducted by Hamdoon Tsai, Yang, and Zhou, the most frequently criticized characteristic is the limited penetration depth (12, 16, 27, 31); which makes it impossible to analyze deeper tissue layers, a prerequisite for an accurate diagnosis. This would lead to an underestimation of the tumor staging (39).

A second aspect that has been analyzed in clinical practice is the possible use of OCT in surgical resection; the Hamdoon (16) study reported a sensitivity and specificity of 81.50% and 87% respectively for the ability of OCT to differentiate between tumor-free and tumor-involved surgical margins. The same level of accuracy was reported by Jerjes (17), who showed that the highest correlation was at 24 hours after resection (r = 0.964). In the buccal mucosal resection margins, OCT and histopathological measurements showed a much better correlation (r = 0.971) compared to other anatomical sites (floor of the mouth, soft palate, lateral tongue and ventral tongue), while the lowest correlation was found for the lower lip (r = 0.578).From Jerjes’ study, it also emerges that there is an underestimation of epithelial thickness: OCT tends to slightly underestimate epithelial thickness, with an average of 20µm in tumor-free margins and 10µm in tumor-involved margins (17). In the Sunny study, another limitation is that OCT has a limited penetration depth (approximately 2 mm), which presents a challenge in assessing deep margins (24). It is also worth noting that numerous studies (12, 16, 17, 23, 27) have used an OCT system to scan ex vivo oral tissues from tumor sections or biopsies, and compared the diagnoses made by OCT with those made by histopathological analysis. However, ex vivo studies do not consider potential motion artefacts, or the anatomical constraints associated with the clinical use of such devices.

### 4.2 Heterogeneity among the methodologies: human versus artificial intelligence

One of the major issues in the review of the literature regarding the use of this device in clinical practice is the great heterogeneity between studies (12, 16, 17, 20, 22–27, 30, 32) in the methods of interpretation of OCT-based imaging.

In the studies conducted by Zhou, Wilder-Smith, Tsai,Panzarella,Jerjes, Obade and Lee (12, 23, 25, 26, 30) the images obtained with OCT were compared with histological examination by two different clinicians, measuring the results obtained according to 5 parameters: sensitivity, specificity, positive predictive value (PPV), negative predictive value (NPV) and accuracy.

Whereas, in the studies conducted by Heidari, Yuan, Yang, Sunny, and James (21, 24, 27, 32) the obtained images were interpreted using two models: the Artificial Neural Network (ANN) and Multi-Level Deep Residual Learning (MDRL). According to the study conducted by Heidari (20) Convolutional Neural Networks (CNNs) are powerful because they can identify distinct features within an image that yield the highest accuracy in classification. Also the paper of James et all (21) showed that OCT images analyzed by automated image processing algorithm (ANN-based analysis) could distinguish dysplastic-OPML and malignant lesions with a sensitivity of 95% and 93%, respectively. Sunny et al. (24) reported a potential clinical application using an artificial intelligence-based algorithm to identify tumor margin areas with 100% accuracy, achieving diagnostic results equivalent to histology (kappa, κ = 0.922); However, its inability to interrogate tissue over 2 mm depth has also been highlighted.

More recent studies (27, 28) have reached the same conclusions, stating that neural networks, both ANN and MDRL, can be used in clinical settings with an excellent diagnostic performance 99.04%,/91.2% sensitivity, 98.81%/83.6% specificity, 98.63/87.5% accuracy respectively (**Table 3**).

**Table 3:** Limitation and method of imaging evaluation.

ID	YEAR	Limitation in clinical practice	Human/Artificial Intelligence (AI)	Strength point	Weaknesses point
*Hamdoon*	2015	Limited depth of tissue penetration	Human	Good sensitivity (81.5%) and specificity (87%) for resection margins. Strong reproducibility.	Ex vivo study. Limited sample size (28 patients).
*Heidari* (20)	2020	Small sample of the true margin can be evaluated	AI	Used AI (CNN) to enhance classification. High sensitivity (100%) and volumetric imaging.	Lower specificity (70%). Small sample (9 patients). No long-term follow-up.
*James* (21)	2021	Limitations associate at human clinical practice	AI	Large sample (347 lesions). High sensitivity (95% for OPMLs, 93% for OSCC). AI-based analysis.	No long-term follow-up. AI models need further validation.
*Jerjes* (17)	2019	Possible error in the use of *ex vivo* OCT concerns variability of the refractive index	Human	60 OSCC patients. High sensitivity (92%) and specificity (94%).	Ex vivo study limits direct clinical application. Potential underestimation of epithelial depth.
*Lee* (22)	2012	(1)Limited applicability to clinical cases: The proposed analysis is only usable when the boundary between the epithelium (EP) and lamina propria (LP) is still present; (2)Limitation in fully automating the diagnostic process;	Human	Computer-aided OCT analysis. Sensitivity (82%) and specificity (90%) for dysplasia.	No clinical validation. Did not test AI-based segmentation.
*Obade* (23)	2021	(1) Need to calibrate clinicians to read and interpret the images, (2) discomfort during the examination process and (3) initial high cost of the scanning tool compared with other commercially available adjunctive tools or low-cost vital staining techniques.	Human	Strong inter-observer agreement. Identified key biomarkers for OSCC detection.	Ex vivo study. No longitudinal follow-up. Variability in feature interpretation
*Panzarella* (30)	2024	OCT interpretation remains highly dependent on the operator’s expertise, and no standardized OCT parameters for diagnosing have been established.	Human	High sensitivity (98.57%) and specificity (100%). Strong inter-observer agreement.	Small sample size (21 patients). Single-center study.
*Sunny* (24)	2019	Inability to interrogate tissue over 2 mm depth	AI	100% sensitivity in intraoperative OCT for surgical margin assessment. Automated diagnostic algorithm.	Small sample (14 patients). Moderate specificity (68.8%).
*Tsai* (25)	2008	Limited depth of tissue penetration	Human	Quantitative OCT approach. High sensitivity and specificity for OSCC detection.	No clinical validation. No longitudinal data. Older technology.
*Wilder- Smith* (26)	2009	Extensive clinical experience is necessary to perform these examinations adequately	Human	One of the earliest *in vivo* OCT studies. Strong inter-observer agreement.	Older technology. No automated image interpretation. Small sample (50 patients).
*Yang*	2021	Limited depth of tissue penetration	AI	Swept-source OCT (100 kHz). AI-assisted classification (98.17% accuracy).	Ex vivo study. Small sample (19 patients). Limited AI model validation.
*Yuan*	2021	Requirement of high-quality images for the AI	AI	Deep learning-based OCT analysis. Sensitivity (91.2%), specificity (83.6%).	Small dataset (37 patients). No real-time validation. Possible AI bias.
*Zhou* (12)	2024	Limited depth of tissue penetration	Human	High sensitivity (93%) and specificity (94%). Histopathological validation.	Ex vivo samples. Small dataset (18 OSCC cases). No AI analysis.

## 5 Limitations

This study has many limitations. One of the main limitations is the heterogeneity of the study designs, as the included studies differ significantly in their methodological approaches. Some are prospective while others are retrospective, leading to potential biases in data collection and interpretation. In addition, the comparison between *in vivo* and ex vivo studies introduces further variability, as differences in tissue handling and imaging conditions may affect the reliability and consistency of reported findings. Another critical limitation is the variability in the anatomical regions analyzed between studies. Different sites within the oral cavity (e.g. tongue, buccal mucosa, floor of the mouth) have different histological and structural characteristics that may influence the optical coherence tomography (OCT) signal and its interpretation. In addition, the lack of studies with complete data sets is a significant barrier to drawing definitive conclusions. Many studies do not report key patient demographics such as age and gender. The inconsistencies in data availability and reporting further complicate efforts to establish standardized diagnostic criteria and guidelines for the clinical use of OCT in oral oncology. Addressing these limitations in future research through standardized study protocols, comprehensive data collection and consistent reporting criteria will be essential to improve the reliability and applicability of OCT in clinical practice.

## 6 Conclusions

OCT holds significant potential for clinical applications, such as guiding biopsy site selection, monitoring lesions, and serving as a rapid, cost-effective screening tool for high-risk populations.

However, the currently available evidence in the literature remains highly heterogeneous, ranging from differences in study methodologies (*in vivo* vs. ex vivo) to variability in the anatomical sections analyzed. Further studies and standardization are certainly necessary for an accurate diagnosis.

OCT has demonstrated high diagnostic accuracy in differentiating between normal, dysplastic and malignant tissue, with sensitivity and specificity often exceeding 80-90%. In addition, OCT’s ability to assess basement membrane integrity has been recognized as a key factor in distinguishing OSCC from other oral conditions. However, several limitations remain, in particular the limited penetration depth of the technology, which limits its ability to analyze deeper tissue layers. This limitation could lead to underestimation of tumor staging and challenges in surgical margin assessment, as highlighted by several studies. In addition, operator-dependent image quality and underestimation of epithelial thickness further complicate its clinical application. Advanced algorithms, including machine learning, deep learning, and artificial intelligence, have demonstrated significantly higher diagnostic sensitivity and, in many cases, greater diagnostic accuracy compared to clinician expertise, but still not reliable enough Moreover, the increasing reliance on such adjuncts could potentially reduce clinicians’ expertise in interpreting OCT images.
